# Determinant role for the *gep* oncogenes, Gα12/13, in ovarian cancer cell proliferation and xenograft tumor growth

**DOI:** 10.18632/genesandcancer.72

**Published:** 2015-07

**Authors:** Ji Hee Ha, Rohini Gomathinayagam, Mingda Yan, Muralidharan Jayaraman, Rajagopal Ramesh, Danny N. Dhanasekaran

**Affiliations:** ^1^ Stephenson Cancer Center and the Department of Cell Biology, The University of Oklahoma Health Sciences Center, Oklahoma City, OK, USA; ^2^ Stephenson Cancer Center and the Department of Pathology, The University of Oklahoma Health Sciences Center, Oklahoma City, OK, USA

**Keywords:** G proteins, ovarian cancer, Gα12, Gα13, gep, gip2, oncogenes

## Abstract

Recent studies have shown that the gip2 and gep oncogenes defined by the α-subunits of Gi2 and G12 family of G proteins, namely Gαi2 and Gα12/13, stimulate oncogenic signaling pathways in cancer cells including those derived from ovarian cancer. However, the critical α-subunit involved in ovarian cancer growth and progression *in vivo* remains to be identified. Using SKOV3 cells in which the expressions of individual Gα-subunits were silenced, we demonstrate that the silencing of Gα12 and Gα13 drastically attenuated serum- or lysophosphatidic acid-stimulated proliferation. In contrast, the invasive migration of these cells were reduced only by the silencing of Gαi2 or Gα13. Analyses of the xenograft tumors derived from these Gα-silenced cells indicated that only the silencing of Gα13 drastically reduced xenograft tumor growth and prolonged the survival of the mice. Similar, but albeit reduced, effect was seen with the silencing of Gα12. On the contrary, the silencing of Gαi2 or Gαq failed to exert such effect. Thus, our studies establish for the first time that Gα12/13, the putative gep oncogenes, are the determinant α-subunits involved in ovarian cancer growth *in vivo* and their increased oncogenicity can be correlated with its ability to stimulate both proliferation and invasive migration.

## INTRODUCTION

Detection of ovarian cancer at the early stages is still a challenge, and only about 15% of the patients get to be diagnosed at the earliest. Alarmingly, about 61% of the ovarian cancer patients are diagnosed at the therapeutically challenging, metastasized stages of cancer, leading to a poor prognosis and survival. The 2015 NCI, SEER statistics indicates that approximately 21,290 women will newly be diagnosed with ovarian cancer and about 14,180 ovarian cancer patients will die of the disease [1] Such statistical evidence underscore the need for the identification of better diagnostic, prognostic and therapeutic targets for the management of ovarian cancer.

Tumor genesis and progression are mediated by aberrant, and asynchronous signaling networks involving multitudes of receptors and their downstream signaling nodes. Oncogenic signaling nodes involving receptor tyrosine kinases and cytokines are well characterized to a large extent. However, only quite recently, the oncogenic potential of Gα-subunits that primarily transmit signaling from their cognate G protein coupled receptors is beginning to be realized. Tumor promoting activities of GPCRs such as those of thrombin [2], Sphingosine-1-phosphate [3, 4], Prostaglandins [5], and lysophosphatidic acid (LPA) have ben shown to be associated with the activation of specific α-subunits [6] [7][8, 9]. These α-subunits, especially those of Gαi- and Gα12-family, have also been identified to transduce signaling from chemokine family of GPCRs to diverse oncogenic responses underlying tumor promotion and metastasis [10-15] [16, 17]. Due to intrinsic potential of activating tumorigenic pathways, the activated forms of Gαi2 and Gα12/13 subunits are referred to as *gip2* and *gep* oncogenes [18]. In addition to transmitting signals from GPCRs, it has also been identified that these α-subunits act as critical signaling hubs to transduce growth promoting activities from receptor tyrosine kinases [19-22], wnt signaling [22, 23], sonic hedgehog signaling [24-28], hippo signaling [28, 29], and steroid hormone receptors [30, 31]. While these studies point to the potential role of Gα-subunits in cancer genesis and progression, the identity of specific α-subunit(s) involved in promoting tumor growth in a defined cancer context remains to be established.

In such a scenario, it is significant to note that ovarian cancer patients show elevated levels of LPA and the resultant aberrant signaling by LPA-receptors (LPARs) has been correlated with increased cell proliferation, migration, and neovasculogenesis in cancer [32-34]. While these *in vitro* observations clearly implicate specific Gα-subunit(s), downstream of LPA-LPAR signaling, the identity of the Gα-subunit that could promote tumor growth *in vivo* has not been defined. Although Gαi-, Gαq-, and Gα12-family of proteins have been shown to transduce mitogenic as well as motogenic signals from LPARs in ovarian cancer cells [4, 35, 36], a comparative analysis to identify the Gα-subunit involved in stimulating ovarian cancer growth *in vivo* has not been undertaken until now. Therefore, in the present study, we examined the role of Gαi2, Gαq, Gα12, and Gα13 in ovarian cancer cell proliferation and migration *in vitro* and their tumorigenic role in xenograft tumors *in vivo*, by utilizing SKOV3 cells expressing nonspecific scrambled shRNA (SKOV3 NS) control cells and the respective Gα-silenced SKOV3 cells (shGαi2, shGαq shGα12, and shGα13). Our results demonstrate that the silencing of Gα12 and Gα13 attenuates LPA-mediated proliferation of SKOV3 cells, thus establishing a mitogenic role for these α-subunits. We demonstrate further that the migratory potential and the invasive migration of these cells are reduced upon the silencing of Gαi2 and Gα13 and not by Gαq or Gα12. *In vivo* analyses of xenograft tumor growth results indicate that the silencing of Gα13 drastically reduced xenograft tumor growth and prolonged survival of the mice. While the silencing of Gα12 exerts a similar, but rather slightly reduced effect, the silencing of Gαi2 or Gαq failed to show any protective advantage for the tumor bearing mice. Thus, our studies establish for the first time that Gα13 is the primary α-subunit involved in accelerating ovarian cancer growth, *in vivo*, and the increased oncogenicity of Gα13 can be correlated with its ability to costimulate the signaling nodes involved in proliferation and invasive migration.

## RESULTS AND DISCUSSION

**Figure 1:**
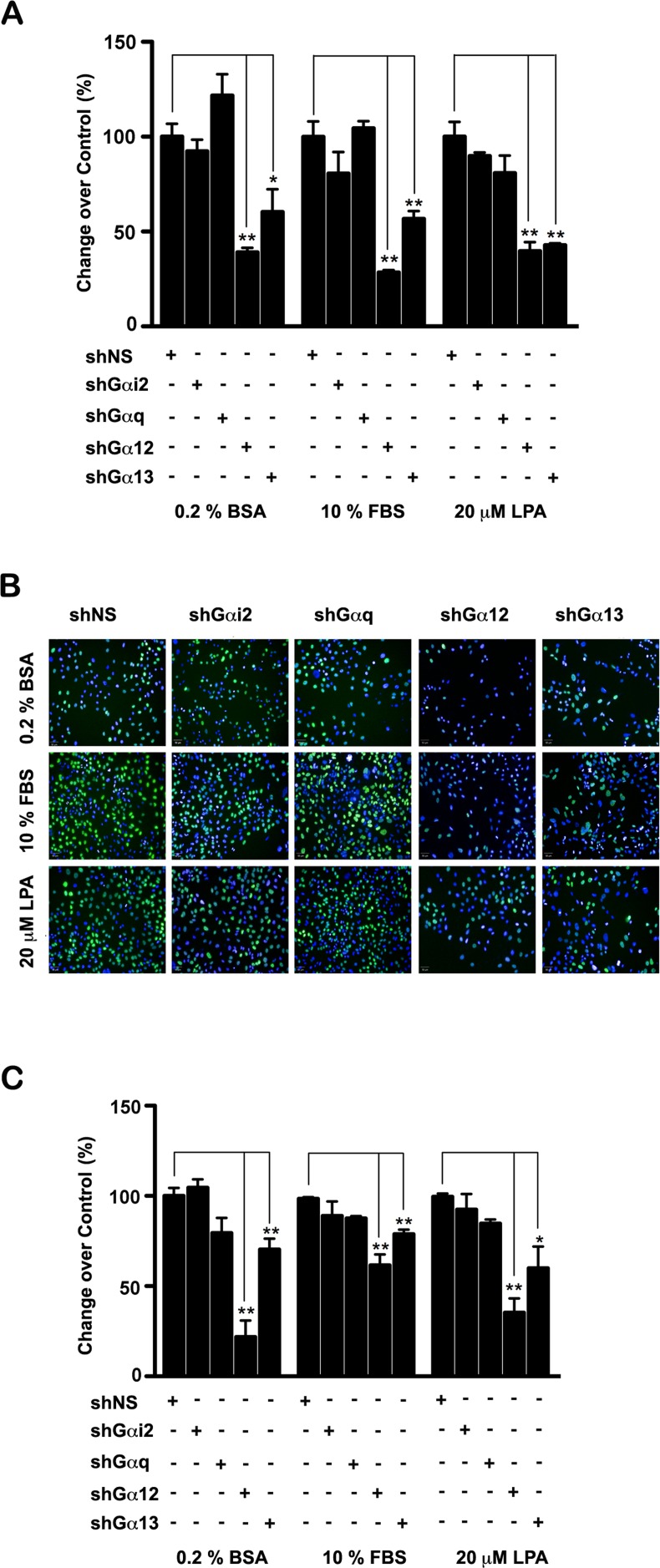
Effect of silencing Gα-subunits in the proliferation of SKOV3 cells **(A)** SKOV3 in which the individual Gα-subunits were silenced (shGαi2, shGαq, shGα12, or shGα13) were plated in 96-well plates (5×10^3^ cells/well) along with the cells expressing scrambled shRNA (shNS). Cell were serum starved for 18 hours, and stimulated with 10% FBS or 20 μM LPA. Cell numbers were determined at 48 hours by live cell imaging in an Operetta HCS imaging analyzer by digital phase contrast imaging and cell count analysis using the Harmony image analysis software. Results are presented as the percent change over control values using the values derived from cells expressing scrambled shRNA as the control. Experiment was repeated thrice and the results are presented as mean ±SEM. Significance was calculated by Student's-t test (*p<0.05, **p<0.01). **(B)** SKOV cells expressing scrambled shRNA (shNS) and cells in which the individual Gα-subunit were silenced were plated in a 96-well plate (5×10^3^ cells/well). Serum starved cells were stimulated with 10% FBS, or 20 μM LPA for 48 hrs. Proliferating S-phase cells were imaged using Click-iT Plus EdU Alexa Fluor 488 imaging kit that monitors 5-ethynyl-2′-deoxyuridine incorporation into DNA. Proliferating cells (green) versus the total number of DAPI-labeled cells (blue) were imaged imaged using Operetta HCS system. **(C)** Proliferating cells versus the total number of DAPI-labeled cells were quantified in Operetta System using the Harmony image analysis software. Results are presented as percent change over the shNS control values (Mean ±SEM; n =3; *p < 0.05, ** p<0.01).

Oncogenic phenotypes in cancer cells are manifested by an increase in the rate of cell proliferation along with a heightened migratory and invasive potential. GPCRs such as LPARs, PARs, CXCRs, and CCRs transmit their oncogenic signaling in cancer cells primarily through Gαi2, Gαq, Gα12/Gα13 in a context specific manner. Additionally, in many instances, these Gα-subunits have also been shown to transmit signaling from non-GPCRs family of receptors in a context specific manner [21, 38, 39]. Therefore, defining the oncogenic potential of these α-subunits has become crucial for the development of highly efficient therapeutics for cancer. This is of greater significance in the case of ovarian cancer in which LPA, which can activate all of these Gα-subunits, plays a major role in the mitogenic and motogenic pathways underlying the disease progression. With this reasoning, we sought to evaluate the cell proliferative, migratory, invasive, and/or tumor-promoting potential of each of these α-subunits. First, we investigated the relative ability of these α-subunits to stimulate cell proliferation in an *in vitro* assay. Proliferation of SKOV3 cells in which the expression of a specific Gα-subunit had been silenced was first monitored by an automated cell enumeration assay using Operetta High Content System. Results from this assay indicated that LPA as well as FBS stimulated an increase in cell number by 48 hrs and this was significantly reduced in the Gα12 or Gα13 silenced cells (Figure 1A). We also monitored the proliferation of these cells using a S-phase cell labeling method that measures the incorporation 5-ethynyl-2′-deoxyuridine into DNA. As shown in Figure 1 (A, B, C), results from both the cell count based and the S phase labeling analysis indicated that the proliferation of these cells were drastically affected by the silencing of Gα12 or Gα13. More significantly, silencing of Gαi2 and Gαq failed to have any such inhibitory effect on LPA or serum mediated proliferation of these cells. While the results obtained with the knockdown of Gα12 and Gα13 confirms our previous findings that LPA as well as serum-stimulated proliferation of ovarian cancer cells is primarily mediated by Gα12 and Gα13 [33, 40, 41], the corollary findings that cell proliferation is not affected by the silencing of Gαi2 or Gαq, firmly establishes the unique role of these *gep* oncogenes in LPA and serum mediated proliferation of ovarian cancer cells.

**Figure 2:**
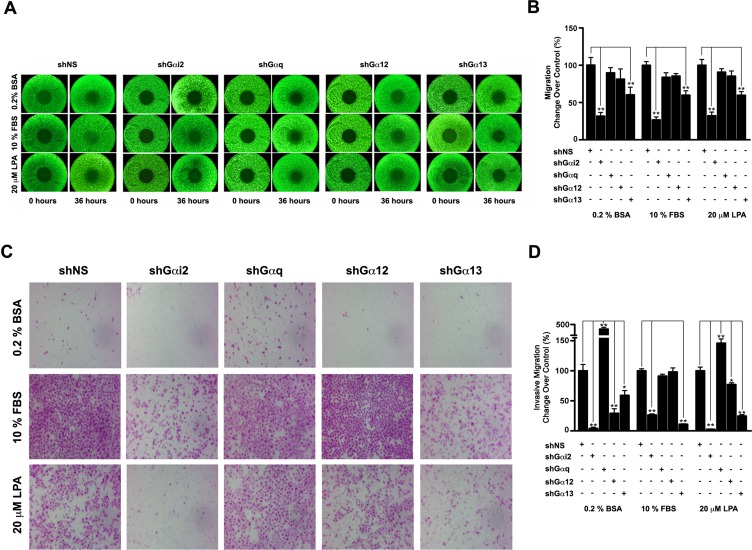
Effect of silencing Gα-subunits on the migration of SKOV3 cells **(A)** SKOV3 cells in which Gai2 (shGαi2), Gaq (shGαq), Ga12 (shGα12), or Ga13 (shGα13) was silenced along with the cells that express scrambled shRNA (shNS) were plated in 96-well Oris TM cell migration plates (3.5 × 10^4^ cells/well) and labeled with cell tracker Green 5-chloromethylfluorescein diacetate (CMFDA) dye. The cells were serum starved for 18 hours (0 hours) and then stimulated with 10% FBS or 20 μM LPA for 36 hours (36 hours). The migration profiles of the transfectants were obtained using the live cell imaging in the Operetta HCS system. The images (2x) presented for 0 hours and 36 hours is a representation of three independent experiments. **(B)** Migrated cells were quantified in Operetta High Content Imaging System using Harmony image analysis software. Results were presented as the percent change in migration over the shNS control values (Mean ±SEM, n = 3). Students-t test was used to obtain the statistical significance and is represented with ** for p<0.01. **(C)** Invasive potential of the respective Ga-silenced cells were monitored using a Transwell migration assay. Cell culture inserts were coated with rat-tail collagen, type 1 and 4 × 10^5^ t NS control, shGα12, shGα13, shGαi2 and shGαq cells were suspended in 200 μl serum-free media and placed in the well of the companion plate. The companion plate wells contained 500 μL of control serum-free media, or serum-free media complemented with 20 μM LPA or 10 % FBS. At the end of 20 hours, the non-migrating cells on the proximal side of the inserts were removed with a cotton swab and the migrated cells on the distal side of the insert were fixed and stained with Hemacolor. Images of migrated cells were obtained from random fields of view at 10X magnification. **(D)** Migrated cells were enumerated SKOV3 cells expressing scrambled shRNA (shNS) and the results were presented as the percent change in invasive migration over the shNS control values with 10% FBS. The values are presented mean ±SEM from three independent experiments. Students-t test was used to obtain the statistical significance and is represented with * for p < 0.05 and ** for p<0.01.

Next, we evaluated the ability of these α-subunits to confer migratory potential to SKOV3 cells, by testing whether the silencing of any of the α-subunits attenuate serum or LPA-mediated migration of these cells. A migration assay using live cell imaging in Operetta High Content Screening system was used to monitor the effect of silencing Gαi2, Gαq, Gα12, and Gα13 upon LPA or serum stimulated migration of SKOV3 cells. Our results indicated that the silencing of Gαi2 or Gα13 drastically reduced LPA/serum-stimulated migration by 68 and 39 % respectively. Silencing of Gα12 and Gαq did not have any such attenuating effect. Thus, contrary to the results obtained with proliferation studies, these results point to a dominant role for Gαi2 and Gα13 in LPA as well as FBS induced cancer cell migration. In addition to an intrinsic increase in migratory potential, cancer cells also exhibit an invasive phenotype. Therefore, we investigated whether Gαi2 and Gα13 have similar effects on the invasive migration of SKOV3 cells. Respective Gα-silenced SKOV3 cells along with scrambled shRNA expressing control cells were evaluated for their ability to inhibit LPA or FBS stimulated invasive migration of ovarian cancer cells, using a collagen-coated Transwell based invasive cell migration assay. Our results indicated that the invasive potential was attenuated significantly again by Gαi2 and Gα13, but not by Gαq or Gα12 (Figure 2B, C). The silencing of Gαi2 attenuated LPA-stimulated cell migration by 97.6 % and FBS-stimulated cell migration by 74.1 %. In a similar fashion, the silencing of Gα13 reduced LPA-stimulated invasive migration it 73.8 % and FBS-stimulated invasive migration by 89.1 %. Although the silencing of Gα12 or Gαq failed to have any effect on FBS-stimulated invasive migration, the silencing of Gα12 reduced LPA-stimulated migration by 19 %. Rather surprisingly, the silencing of Gαq appears to promote overall invasive migration as shown by the serum starved as well as serum-stimulated shGαq cells (Figure 2 C, D). Although the underlying mechanism is not known at present, at least these results rule out a role for Gαq in the invasive migration of ovarian cancer cells. Thus far, there have been contradicting reports on the identity of the Gα-protein involved in LPA- or serum-mediated invasive migration of ovarian cancer cells. In this context, our comparative analysis to identify the role of a specific Gα-protein has finally established that both Gαi2 and Gα13 are involved in promoting cell migration in ovarian cancer cells. It is likely that the spatiotemporal coordination of signaling inputs from both of these α-subunits is involved in the invasive migration of ovarian cancer cells. Further, our observation that Gαi2-Rac as well as Gα13-Rho-mediated pathways are required for LPA mediated invasive migration of ovarian cancer cells [36, 42] as well as the independent findings that the migration mediated by CXCL12-CXCR4 signaling involves both Gαi2-mediated mTORC1 signaling [43] and Gα12/13 mediated Rho signaling pathways [14] supports this view.

**Figure 3:**
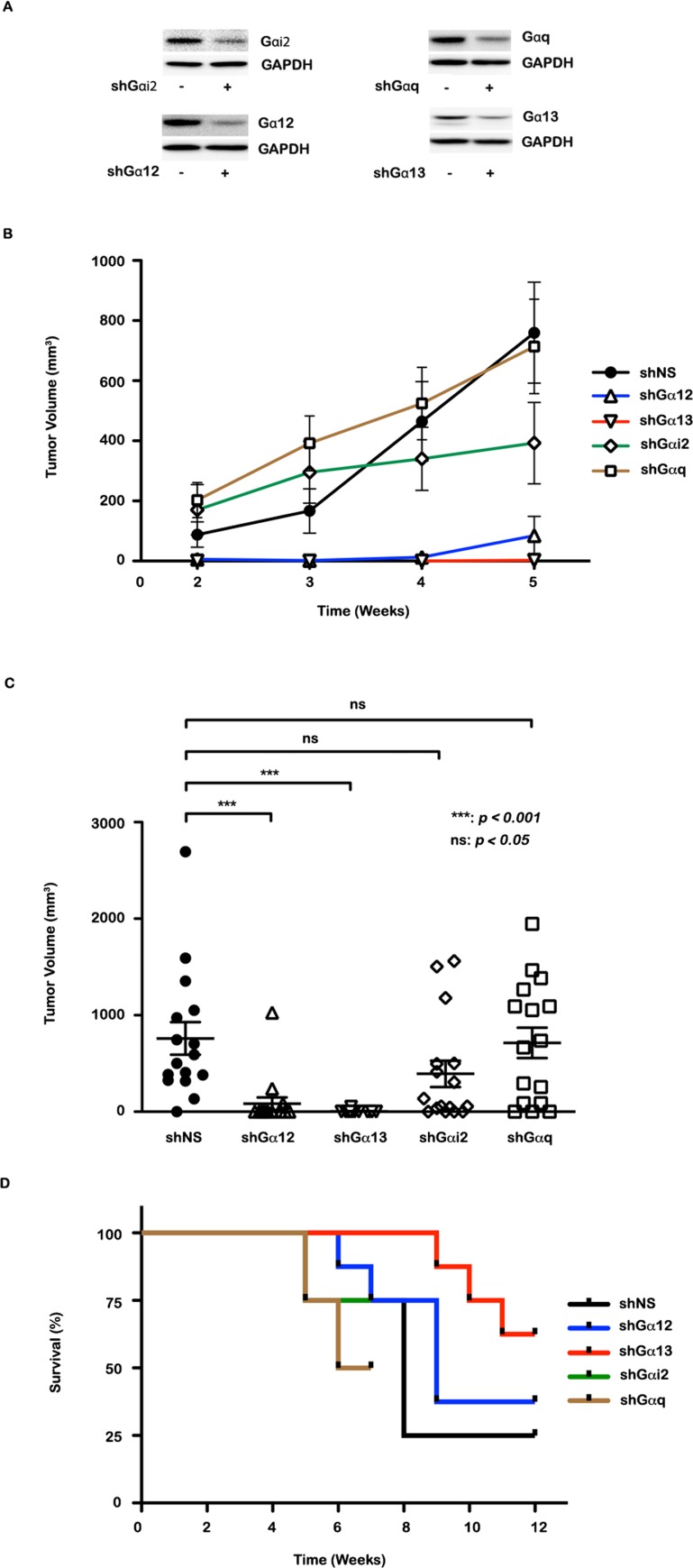
Effect of silencing Gα-subunits on xenograft tumor growth **(A)** Mycoplasma free, stably silenced SKOV3 G protein cell lines (shGα12, shGα13, shGαi2 and shGαq) were ascertained for appropriate G protein silencing by immunoblotting with Gα12, Gα13, Gαi2 and Gαq antibodies and GAPDH antibody for monitoring equal loading of lysate protein. **(B)** 1×10^6^ SKOV3 NS control (nonspecific scrambled control) and SKOV3 G protein silenced (shGα12, shGα13, shGαi2, shGαq) ovarian cancer cells were subcutaneously injected on the dorsal surface of NU/NU nude mice. The tumor volume of all the experimental groups (shGα12, shGα13, shGαi-2, shGαq) were measured over a period of five weeks and assessed in comparison to the NS control group. **(C)** Mean tumor volumes and individual tumor volumes of each group were assessed between NS control and G protein silenced groups. Statistical significance for tumor volume differences and mean tumor volumes between the NS control group and the experimental groups were determined using students-t test, *p<0.05, **p<0.01. **(D)** The percentage of survival of each G protein silenced group (shGα12, shGα13, shGαi2, and shGαq) was determined with respect to the control group, by monitoring their survival for a period of 12 weeks.

Together with the data on cell proliferation, our results demonstrate that Gα12 and Gα13 are involved in stimulating cell proliferation with no significant role for Gαi or Gαq. However, invasive migration of ovarian cancer cells appears to be dependent on Gαi2 and Gα13. All of these α-subunits have been implicated in the tumorigenesis and tumor progression in many cancers [29, 34, 44-46]. Therefore, we sought to investigate the role of these Gα-subunits in ovarian cancer growth *in vivo* by using the respective Gα-silenced xenograft tumor mice model. Results from the mice bearing xenograft tumors of the Gα-silenced ovarian cancer cells, namely shGαi2-SKOV3, shGαq-SKOV3, shGα12-SKOV3, and shGα13-SKOV3, indicate that the control, Gαq and Gαi2 silenced SKOV3 tumors in mice exhibited exponential growth in tumor volume over a period of five weeks (Figure 3B & 3C). More significantly, a decrease in tumor volume was observed in Gα12 and Gα13-silenced SKOV3 xenograft tumors in comparison with the other groups (Figure 3B, C). In this regard, our findings agree to a certain extent with the results obtained with non-small cell lung carcinoma cells, in which the silencing of either Gα12 or Gα13 independently decelerated tumor growth [47]. However, our analysis of animal survival data using Kaplan-Meier plot indicated a more significant role for Gα13 than any other α-subunits. As shown in figure 3C, the silencing of Gα13 prolonged the survival of the xenograft tumor bearing mice compared to the ones bearing shGαi2, Gαq, or control group. Although, the silencing of Gα12 was not as protective as Gα13, these animals also showed extended survival compared to Gαi2, Gαq, or NS-silenced xenograft tumor bearing mice. In fact, the animals bearing Gαq-silenced xenograft tumor showed aggressive tumor growth that led to the euthanization of the animals as early as 7 weeks. Thus, our studies point to a critical role for Gα13 in promoting tumor progression *in vivo*, especially in ovarian cancer context. Nevertheless, it should be noted here that silencing of Gα12 also led to tumor growth inhibition next only to the silencing of Gα13. Interestingly, the differences in the tumor volumes and tumor growth between shGα12 and shGα13 tumor group animals were quite minimal, which is consistent with the findings that they show 67% amino acid identity and they are involved in the activation of many similar pathways. However, Gα12 and Gα13 are also involved in the activation of unique pathways of their own. Thus, it is possible that the increased protective effect of Gα13 on tumor bearing animals, compared to Gα12, may be indicative of the unique pathways activated by Gα13. Quantitatively, our results differ by establishing a more dominant role for Gα13. In addition, by assessing the role of other growth promoting Gα-subunit in ovarian cancer context in which aberrant signaling by LPARs play a critical role, we firmly establish a determinant role for the *gep* oncogenes represented by Gα12 and Gα13 and not to Gαi2. A simple paradigm based on the results presented here along with our previous data would suggest that the increased oncogenicity of Gα13 could be associated with its unique ability to stimulate both mitogenic and motogenic pathways. Although mitogenic pathways and motogenic pathways are often mutually exclusive, it is possible that Gα13-mediated effects, on both of these signaling events are temporally regulated. Considering the heterogeneity of tumor cell population in cancer tissue, it is also possible that Gα13 stimulates proliferation or migration in different population of cancer cells, which collectively contribute towards aggressive tumor growth. Further defining of the unique downstream signaling nodes associated with Gα13 signaling axis could unravel novel targets for ovarian cancer therapy and disease management.

## MATERIALS & METHODS

### Cell lines and culture

Control SKOV3 cell expressing nonspecific scrambled shRNA (SKOV3 NS) and the Gα-silenced SKOV3 cell lines (shGαi2, shGαq, shGα12, and shGα13) were cultured and maintained in Dulbecco's modified Eagle's Medium (DMEM) (Cellgro, Manassas, VA), containing 10% Fetal Bovine Serum (Gemini Bio-Products, West Sacramento, CA), 50 units/mL Penicillin, and 50 μg/mL Streptomycin at 37°C in a 5% CO2 incubator. Oleoyl (18.1) LPA was obtained from Avanti Polar Lipids (Alabaster, AL) was dissolved in PBS containing 0.1% BSA as 10 mM stock solutions, and stored at −20°C.

### Stable Cell line Generation and Immunoblot analysis

Non-target control shRNA pLKO.1 vector construct was purchased from Sigma-Aldrich, St. Louis, MO (SHC002) whereas pLKO.1 vector constructs targeting Gαi2 (RHS3979-9596925), Gαq (RHS3979-9604171), Gα12 (RHS3979-98491914), and Gα13 (RHS3979-9604295) were purchased from Open Biosystems (Lafayette, CO). Stable transfections were performed using Amaxa Biosystems Nucleofector II, according to the instructions of the manufacturer. The stably transfected NS control and Gα-silenced clones were selected with puromycin (2 μg/ml; MP Biomedicals, Solon, Ohio) and single clones were picked, expanded to obtain stable cell lines. The efficiency of silencing the expression of the respective α-subunit was ascertained in the respective stable cell lines using immunoblot analysis, in accordance to our previously published methods [37]. Antibodies to Gαi2 (sc-13534), Gαq (sc-393), Gα12 (sc-409), Gα13 (sc-410), were purchased from Santa Cruz Biotechnology Inc, CA, and the GAPDH antibody was purchased from Life Technologies-Ambion (AM4300). Peroxidase-conjugated anti-rabbit IgG (W401B) and anti-mouse IgG (NA931V) were purchased from Promega Corporation (Madison, WI). The blots were developed using SuperSignal West Pico chemiluminescent substrate (34080) from Perkin Elmer (Waltham, MA) and imaged using Kodak Image Station 4000 MM.

### Cell Proliferation Assays

Cell proliferation in Gαi2-, Gαq-, Gα12-, or shGα13-silenced SKOV3 cells along with the control NS SKOV3 cells was monitored by two different assays. A cell count based assay was carried out in which the increase in cell number following the stimulation with 20 μM LPA or 10% FBS. Respective Gα-silenced SKOV3 cells (5×10^3^ cells) along with nonspecific scrambled shRNA expressing control cells were plated in 96-well plates, serum starved for 18 hours, and then stimulated with 10% FBS or 20μM LPA. Images were obtained at 0, 24 and 48 hours using digital phase contrast imaging in Operetta High Content Imaging System and quantified by Harmony, a high content imaging and analysis Software. To monitor the extent of cell proliferation, the percentage of proliferating cells (S-phase cells) was analyzed by deoxynucleotide (5-ethynyl-2′-deoxyuridine or EdU) incorporation assay, using a Click-iT Plus EdU Alexa Fluor 488 imaging kit (C10637) from Life Technologies (Grand Island, NY). Briefly, 5×10^3^ cells of each cell line was plated in 96 well plates, serum starved, stimulated with 10% FBS or 20μM LPA and incubated with 10μM EdU at 22 hours. After 2 hours of incubation, the EdU incrporated cells were fixed, permeabilized, stained according to the manufacturer's protocols. Proliferating cells versus the total number of DAPI-labeled cells were imaged and quantified in Operetta High Content Imaging System using Harmony, the built-in image analysis software.

### Cell Migration Assay

Migratory potential was monitored using Oris Cell Migration assay (Platypus Technologies. Madison, WI, # CMACC5.101). 35 × 10^3^ cells Gα-silenced SKOV3 cells, along with the control cells were labeled with Cell Tracker Green CMFDA Dye (Life technologies-Molecular Probes, #C2925) and plated in 96-well Oris TM cell migration plates (CMACC5.101) using manufacturer's protocol. Briefly, the transfectants were serum starved for 18 hours. and the detection zone blocking stoppers were removed. Cells were treated for 1h with 0.5 μM mitomycin (47589, Calbiochem, La Jolla, CA) to inhibit proliferation and then stimulated with 10 μM LPA or 10% FBS for 36 hours. Pre-migration (0 hr) and migration of cells into the detection zone at 36 hrs were imaged using Operetta High Content Imaging System. Migrated cells were quantified by Harmony image analysis software of the Operetta and the percentile cell migration at 36 hrs over the 0-hr controls were plotted.

### Invasive Migration Assay

Invasive migration assay was carried in accordance to our previously described [33, 37] methods. Cell culture inserts (polyethylene terephthalate membrane with 8.0 μm pores #353097, BD Biosciences, Franklin Lakes, NJ) were coated with rat-tail collagen, type 1 (BD Biosciences) and 4 × 10^5^ cells suspended in 200 μL serum-free media were placed in the well of the companion plate. The companion plate wells contained 500 μL of control serum-free media and either serum-free media with 10 μM LPA or 10 % FBS. At the end of 20 hours, the non-migrating cells on the proximal side of the inserts were removed with a cotton swab and the migrated cells on the distal side of the insert were fixed and stained with Hemacolor (EMD Chemicals, Inc., Gibbstown, NJ). The migrated cells were enumerated with the images obtained from random fields of view at 10X magnification and the results were presented as the percentage of migration.

### Animal experiments and ethical compliance

Nu/Nu nude mice (5-6 weeks old) were purchased from Charles River laboratories (Wilmington, MA) and were housed in a barrier facility under 12hour light/dark cycle under pathogen free conditions, with food and water *ad libitum*. All experiments were performed with the approval of the university of Oklahoma Health Science Center institutional animal care and use committee. Mycoplasma free NS-SKOV3 (nonspecific scrambled shRNA control) and shGαi2-SKOV3, shGαq-SKOV3, shGα12-SKOV3, or shGα13-SKOV3 cells (1×10^6^) were injected subcutaneously on the dorsal surface of NU/NU nude mice to obtain ovarian cancer xenograft tumors. The control and experimental groups were monitored regularly for tumor development, and the tumor volume in all the groups were measured for over a period of five weeks. Animals experiencing pain or cachexic symptoms were euthanized appropriately with the opinion of the institutional veterinarian.

### Statistics

Graph pad prism software (La Jolla, California) was utilized to perform Student's t-test and Kaplan–Meir analysis.
